# Distribution of TAS2R38 bitter taste receptor phenotype and haplotypes among COVID-19 patients

**DOI:** 10.1038/s41598-022-10747-2

**Published:** 2022-05-05

**Authors:** D. Risso, D. Carmagnola, G. Morini, G. Pellegrini, E. Canciani, M. Antinucci, D. Henin, C. Dellavia

**Affiliations:** 1 Tate & Lyle PLC, 5 Marble Arch, London, W1H 7EJ UK; 2 Department of Biomedical, Surgical and Dental Sciences, Università degli Studi di Milano, Via Mangiagalli 31, 20133 Milan, Italy; 3 University of Gastronomic Scienceas, Piazza Vittorio Emanuele 9, Bra, 12042 Pollenzo, CN Italy; 4 Department of Medicine and Life Sciences, Institute of Evolutionary Biology (UPF-CSIC), Universitat Pompeu Fabra, Barcelona, Spain

**Keywords:** Genetics, Medical research, Risk factors, Respiratory tract diseases, Viral infection

## Abstract

Bitter taste receptor TAS2R38 is expressed in the respiratory tract and can respond to quorum-sensing molecules produced by pathogens, stimulating the release of nitric oxide, with biocidal activity. TAS2R38 presents two main high-frequency haplotypes: the “taster” PAV and the “non-taster” AVI. Individuals carrying the AVI allele could be at greater risk of infections, including SARS-CoV-2. The aim of this study was to assess the frequency of PAV and AVI alleles in COVID-19 patients with severe or non-severe symptoms compared to healthy subjects to further corroborate, or not, the hypothesis that the PAV allele may act as a protecting factor towards SARS-CoV-2 infection while the AVI one may represent a risk factor. After careful selection, 54 individuals were included in the study and underwent genetic analysis and PROP phenotype assessment. Our investigation could not point out at a significant relationship between single nucleotide polymorphisms responsible for PROP bitterness and presence/severity of SARS-CoV-2 infection, as previous studies suggested. Our results uncouple the direct genetic contribution of rs10246939, rs1726866 and rs713598 on COVID-19, calling for caution when proposing a treatment based on TAS2R38 phenotypes.

## Introduction

The immune system is also referred as the “sixth sense”, being able to detect pathogens in the body, like the other senses detect stimuli in the external environment^1^. The identification of taste receptors (especially of bitter taste receptors) expressed extra-orally, brought to the discovery of their additional involvement in innate immunity^2,3^. In humans, bitter taste is mediated by 25 functional bitter taste receptors (TAS2Rs), belonging to the superfamily of G protein-coupled receptors (GPCRs)^4–6^. In particular, it has been proven that one of them, namely TAS2R38, abundantly expressed in the respiratory tract, is able to respond to quorum-sensing molecules produced by some pathogens, and that its activation stimulates the release of nitric oxide (NO), with biocidal activity^7,8^. NO and its derivatives also cause a reduction in viral RNA production in the early steps of viral replication and of viral protein synthesis, inhibiting the replication cycle of the severe acute respiratory syndrome coronavirus (SARS-CoV), through the reduction in the palmitoylation of nascently expressed spike (S) protein which affects the fusion between the S protein and its cognate receptor, angiotensin converting enzyme 2 (ACE-2)^9,10^.

TAS2R38 presents several variants, with two main global high-frequency haplotypes in worldwide populations, characterized by a different capability to recognize the chemically similar artificial compounds 6-n-propylthiouracil (PROP) and phenylthiocarbamide (PTC): the “taster” PAV (encoding proline, alanine, and valine at the respective variant sites) and the “non-taster” AVI (encoding alanine, valine, and isoleucine at these sites)^11^. They are formed by three single nucleotide polymorphisms (SNPs) in high (> 0.9) linkage disequilibrium (LD), namely rs713598, rs1726866, and rs10246939, at positions encoding amino acids 49, 262 and 296, respectively, that account for more than 70% of the variation in taste sensitivity to this compound^12^. Although the ability to taste PTC and PROP is inherited in a nearly Mendelian recessive manner, the correlation between TAS2R38 haplotypes and phenotypes, although strong, is not perfect and a small fraction of such variability is still not understood^11,12^. Indeed, considering the discovery of extra-oral bitter taste receptors, it has been suggested that forthcoming approaches to TASRs studies should examine a wide spectrum of both taste and non-taste phenotypes to better assess the significance of their variability^13^. Interestingly, the non-taster allele is not responding also to quorum sensing molecules^7^, with the consequence that individuals carrying the AVI allele could be at greater risk of infections.

The role of bitter taste receptors in the response to pathogens of the respiratory tract brought some researchers to raise the possibility that bitter taste receptors could represent therapeutic targets for the clinical symptoms of severe acute respiratory syndrome Coronavirus strain 2 (SARS-CoV-2)^14^. Since TAS2R38 has been identified as one of the most involved receptors in the innate immune response of the respiratory tract and thanks to the ease in identifying its main polymorphisms through the tasting of PTC strips, some retrospective studies have investigated the correlation between T2R38 phenotype and COVID-19 symptoms severity^15^ and duration^16^. Both reported that non-tasters were significantly more likely than tasters and supertasters to test positive for SARS-CoV-2, to be hospitalized once infected, and to be symptomatic for a longer duration, suggesting enhanced innate immune protection against SARS-CoV-2.

Moreover, it has been proposed that assessing treatment protocols for COVID-19 patients according to their TAS2R38 phenotype could provide additional value to achieve a successful treatment and possibly other respiratory tract pathogens^17^.

However, no TAS2R38 genotypic data were reported in these studies. Similarly, a paper analyzing in silico data on the allele frequency and COVID-19 death rate in different countries highlighted how a higher presence of the TAS2R38 PAV allele than AVI is associated with lower COVID-19 mortality^18^. A big limitation of this investigation, however, is that only a causal relationship could be reported.

To date no papers have investigated the frequency of PAV and AVI alleles via genetic analysis in COVID-19 patients with different severity of the diseases and healthy subjects. The aim of the present study was therefore to preliminary assess the frequency of PAV and AVI alleles in COVID-19 positive patients with severe or non-severe symptoms compared to COVID-19 negative subjects to further corroborate, or not, the hypothesis that the PAV allele may act as a protecting factor towards SARS-CoV-2 infection while the AVI one may represent a risk factor.

## Materials and methods

### Subjects

The study complied with the principles of the Declaration of Helsinki. Ethical clearance for the study was approved by Università degli Studi di Milano (Italy) (number 36/21, 20.04.2021). General inclusion criteria for participating included age > 18 years and Caucasian ethnicity (in order to limit genetic variability).

**Figure 1:**
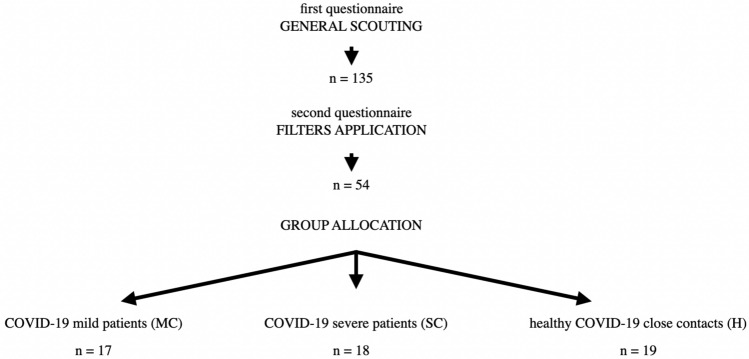
Target population selection process.

The target population was identified through a process outlined in Fig. 1. By means of a first questionnaire, sent by e-mail, explaining the overall goal of the study and including questions on personal information and data related to COVID-19 experience (i.e., if the subjects had had COVID-19, if symptomatic or asymptomatic, or if they did not contract COVID-19 despite sharing facilities with positive household fellows), a preliminary study population was sounded by word of mouth among staff, relatives and acquaintances of the researchers group of the This Section Lab of Department of Biomedical, Surgical and Dental Sciences, Università degli Studi di Milano. A total of 135 responding subjects agreed to sign an informed consent and were asked to answer a second questionnaire, sent by e-mail, with more detailed questions on their general health, and, for subjects who had contracted COVID-19, their symptoms and clinical course, in order to assess whether they fulfilled the inclusion criteria for entering one of the 3 following groups:

- COVID-19 mild patients (MC): subjects that had COVID-19 but did not show severe symptoms or any symptoms at all (see inclusion criteria)
- COVID-19 severe patients (SC): subjects that had COVID-19 and showed severe symptoms (see inclusion criteria)
- healthy COVID-19 close contacts (H): subjects highly exposed to the risk of COVID-19 infection but did not contract the infection

To define the SC and MC groups, NIH criteria were used^19^, since this classification quite comprehensive and easily adaptable for self-reporting by the patients was found.

#### COVID-19 subjects inclusion criteria

In general, this group included subjects infected by SARS-CoV-2 and then healed, as proven by a positive real-time reverse transcriptase-PCR assay (RT-PCR) of a naso-pharyngeal swab specimen for SARS-CoV-2 RNA, followed by a negative one.

Further, the severity of COVID-19 symptoms classification^19^, lead to the definition of the following sub-groups:

- MC group, referring to subjects who displayed very mild to moderate illness. More in detail, mild illness referred to individuals who had any of the various signs and symptoms of COVID-19 (e.g., fever, cough, sore throat, malaise, headache, muscle pain, nausea, vomiting, diarrhea, loss of taste and smell) but who did not report shortness of breath, dyspnea, or abnormal chest imaging. Moderate illness referred to individuals who showed evidence of lower respiratory disease during a clinical assessment or imaging and with a saturation of oxygen (SpO2) ≥ 94% on room air at sea level.
- SC group, referring to subjects who presented severe illness according to the following criteria: SpO_2_ < 94% on room air at sea level, a ratio of arterial partial pressure of oxygen to fraction of inspired oxygen (PaO_2_/FiO_2_) < 300 mm Hg, respiratory frequency > 30 breaths/min, or lung infiltrates > 50%.

#### COVID-19 healthy close contacts inclusion criteria

- non-vaccinated subjects that entered in close contact with symptomatic COVID + family members. Close contact was defined as living in the same house and being closer than 1 m without facial mask over 1 day/night.
- negative serologic test for SARS-CoV2 (IgG and IgM antibodies) (Euroimmun Medizinische Labordiagnostika, Lubeck, Germany).

Exclusion criteria:

- pathological lesions of the oral mucosa including lingual dorsum
- systemic diseases and pharmacological treatments for chronic disease.

Each enrolled subject received a kit including a leaflet with simple and clear instructions on how to proceed for self testing and the tools needed for PROP tasting phenotype assessment and biological material collection.

### DNA collection, extraction and sequencing

Each of the subjects included in the study provided a biological sample by means of a sterile cotton swab (Puritan Medical Products, Guilford, ME) that they scrubbed on their buccal mucosa for 3 min in triplicate. The buccal swabs were then air-dried for 2 h and stored at – 20 °C for 1 week until the time of DNA extraction process^20^. Total DNA was extracted from swab using QIAamp DNA Mini kit (Qiagen, Hilden, Germany) according to the manufacturer’s instructions. Amplification reactions were performed with AmpliTaq Gold™ 360 (Applied Biosystems™, ThermoFischer Scientific, Walthman, MA, USA) using primers TAS2R38_For 5′-CCCTCTAAGTTTCCTGCCAGA-3′, TAS2R38_Rev 5′-GCTTTGTGAGGAATCAGAGTTGT-3′ on a Thermal Cycler 5333 MasterCycler (Eppendorf). Briefly, each sample was treated at 95 °C for 5 min to allow the activation of the DNA polymerase, and then DNAs were amplified by 40 three-step cycle (denaturation at 95 °C for 30 s, annealing at 59 °C for 30 s and extension at 72 °C for 70 s) followed by a final extension step of 10 min at 72 °C. PCR products (1291 bp) were then analyzed on a 1.5% agarose gel and then treated with ExoSap (Applied Biosystems™, ThermoFischer Scientific, Walthman, MA, USA). Applied Biosystems Sequencing was performed with BigDye™ Terminator v1.1 Cycle Sequencing Kit (ThermoFisher Scientific, Walthman, MA, USA) on a AB3730xl DNA Analyzer™, (ThermoFisher Scientific, Walthman, MA, USA). The used oligonucleotide primers for sequencing were the same of PCR with the addition of these internal primers: TAS2R38_ForInt 5′-CCCAGATGCTCCTGGGTA-3′ and TAS2R38_RevInt 5′-GATCTTTAATCTGCCAGTTGAGC-3′. Sequences obtained were assembled on the reference sequence and then analyzed with SeqScape™ Software v3.0 (ThermoFisher Scientific).

#### Assessment of PROP phenotype

PROP-phenotype was assessed using cotton swabs dipped in a suprathreshold 50 mM 6-n-propylthiouracil solution (Sigma Aldrich S.r.l.), as previously described^21^. Subjects were asked to hold the cotton PROP swab for 10 s and asked to rate the bitterness intensity on a Labeled Magnitude Scale (LMS^22^).

### Genetic and statistical analyses

#### Phasing

The first aim was to reconstruct the haplotypes for rs10246939, rs1726866 and rs713598 SNPs in each subject in our dataset. To do so, the algorithm PHASE, v.2.1^23,24^ was used, which adopts a Bayesian method to phase genotypic data and can improve its performance upon feeding the algorithm with previously phased samples. To achieve such higher precision, it was used the phased genotypes of the 2504 available samples in the 1000 Genomes Project phase 3^25^ for the three SNPs included our study. When PHASE run with our dataset plus the specified known phases form 1000 Genomes Project, the results showed that each pair of haplotypes in our samples had the highest possible probability, highlighting the accuracy of the analysis. To further test these results, the analysis was performed using another software, SHAPEIT v.2.17^26^, whose results fully corroborated previously phased genotypes. Finally, in order to facilitate association of samples with PROP perception-related diplotypes, each haplotype (GCC—ATG) was converted to the relative amino acid sequence present in TAS2R38 receptor (PAV—AVI).

### Genetic association analyses

Being able to assess a clear and powerful association between TAS2R38 genotypes and PROP perception nowadays has become a quality control when working with such deeply studied data. It was tested for that with PLINK v.1.7 (http://pngu.mgh.harvard.edu/purcell/plink/)^27^ using logistic and linear regression analyses, while controlling for covariates such as age and sex, BMI and smoking status for all samples. Furthermore, SNPs included in our study were also tested with the other available phenotypes, namely dysgeusia status and presence/severity of COVID-19 infection. All analyses run with default parameters and significance were adjusted with Bonferroni correction for multiple tests.

### Statistical analyses

Statistical support for putative differential apportionment of PROP perception scores in COVID-19 infection and dysgeusia groups was assessed via ANOVA. It was also tested whether the distribution of each sample’s diplotype (PAV/PAV, PAV/AVI or AVI/AVI) was found more (or less) than expected by chance across the different COVID-19 status. To do so, in addition to ANOVA, hypotheses testing included Chi-squared test of independence and Fisher exact test.

## Results and discussion

A preliminary scouting allowed us to identify 135 individuals willing to participate, but then a careful selection process was applied aimed to reduce the effect of potential confounding factors on our analysis. Therefore, all subjects with systemic diseases or conditions, or on medications of any kind, were withdrawn, as well as those who filled the first questionnaire poorly and could not be clearly assigned to any of the three groups (COVID-19 mild patients (MC), COVID-19 severe patients (SC), healthy COVID-19 close contacts (H). The strict selection of study population did not take into account potentially important information on systemic disease and medication, however these data were not the topic of the study that was focused on the PAV and AVI alleles and their role as protecting factors in COVID-19 patients.

Concerning the H group (subjects highly exposed to the risk of COVID-19 infection but who did not contract the infection), only subjects actually sharing home facilities with COVID-19 positive patients before knowing and isolating them, were included. At the end of this process, 54 study participants were selected. In particular, 19 subjects qualified for the H group, 17 for the MC and 18 for the SC group.

The 54 enrolled individuals had a mean age of 46 years (range 22–90) and 29 were females (54%). Concerning gender distribution in the 3 groups, the H one counted 12 females (63%), the MC one 9 (53%) and the SC one 8 (44%). Concerning age, the H group showed a mean value (range) of 44 (24–83), the MC group of 45 (26–90) and the SC group of 49 (22–79).

Further relevant data of the 3 groups are described in Table 1.

**Table 1:** Main medical characteristics of the 3 groups.

Group	n	Hospitalized	Days at hospital (mean and range)	Temperature > 37 °C	Generic symptoms^a^	Cough and sore throat	Gastrointestinal symptoms^b^	Pneumonia	Peripheral O_2_ saturation < 94%	Taste and/or smell alterations
H	19	0	0	0	0	0	0	0	0	0
MC	17	0	0	16 (94%)	13 (76%)	11 (65%)	5 (29%)	0	0	12 (71%)
SC	18	9 (50%)	34 (5–90)	16 (89%)	16 (89%)	13 (72%)	12 (67%)	10 (56%)	13 (72%)	11 (61%)

*N* number.

^a^Including headache, fatigue, generic muscle pain; ^b^including nausea, diarrhea, vomiting.

**Figure 2:**
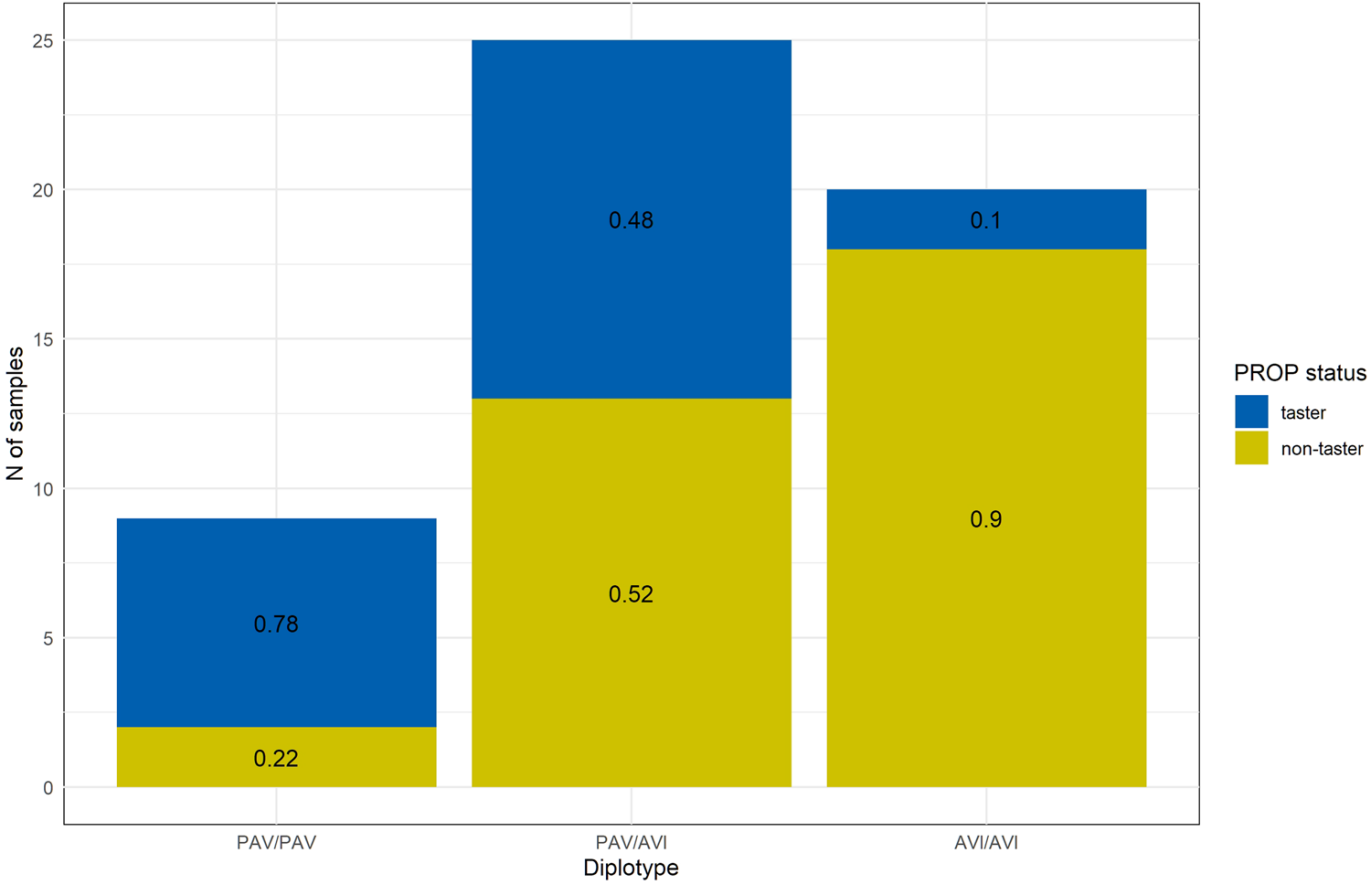
PROP status distribution among TAS2R38 diplotypes.

Our analyses to infer TAS2R38 haplotypes showed that all samples exclusively carried the two most common and widespread sequences of alleles, namely CCG and GTA, respectively coding for PAV and AVI amino acids at positions 49, 262 and 296. The PROP perception score was recorded, assessed in taste test, to obtain the binary variables “taster” and “non-taster” as previously described^21^, thus being able to classify individuals based on their ability to perceive PROP bitterness. Out of 54 total samples, 21 (39%) were classified as non-taster and 33 (61%) as taster, while, regarding TAS2R38 haplotypes, 9, 25 and 20 (17%, 46%, 37%) individuals carried, respectively, PAV/PAV, PAV/AVI and AVI/AVI diplotypes. Moreover, PROP status (i.e. being either taster or non-taster) followed expected distribution across diplotypes, with PAV/PAV, PAV/AVI and AVI/AVI samples respectively showing tasters frequencies of 0.78, 0.48 and 0.1 and non-tasters frequencies of 0.22, 0.52 and 0.9 (Fig. 2).

These trends point once again towards the higher PROP sensitivity of PAV homozygotes, the intermediate position of the heterozygotes and the reduced perception in AVI homozygotes. When considering COVID-19 phenotypes, among the 35 infected individuals (65% of all samples), 21 (60%) reported dysgeusia and 12 (34%) did not, while the two remaining samples could not assess their taste status.

Analyses performed with PLINK confirmed the known association between TAS2R38 SNPs and PROP score reported in the test, highlighting how even in a relatively small dataset, this association still holds true. In particular, linear regression shows how the presence of alleles rs10246939 G, rs1726866 C and rs713598 C is related to higher PROP scores (corrected p value < 0.05), pointing again at PAV haplotype as the “taster” one. This result still maintained significant even when testing SNPs with PROP status, taster and non-taster, via logistic regression. Both the analyses gave insights on how age, used as a covariate, also contributes to PROP bitter perception, indicating a negative association with PROP scores. In this latter case, however, tests did not remain significant after correction. It was also tested whether our SNPs were significantly related to dysgeusia and COVID-19 infection phenotypes. None of the tests performed provided significant results, highlighting how, at least in our dataset, TAS2R38 genotypes are not responsible for differences in the presence or the severity of COVID-19 infection, and do not influence infection-driven dysgeusia.

**Figure 3:**
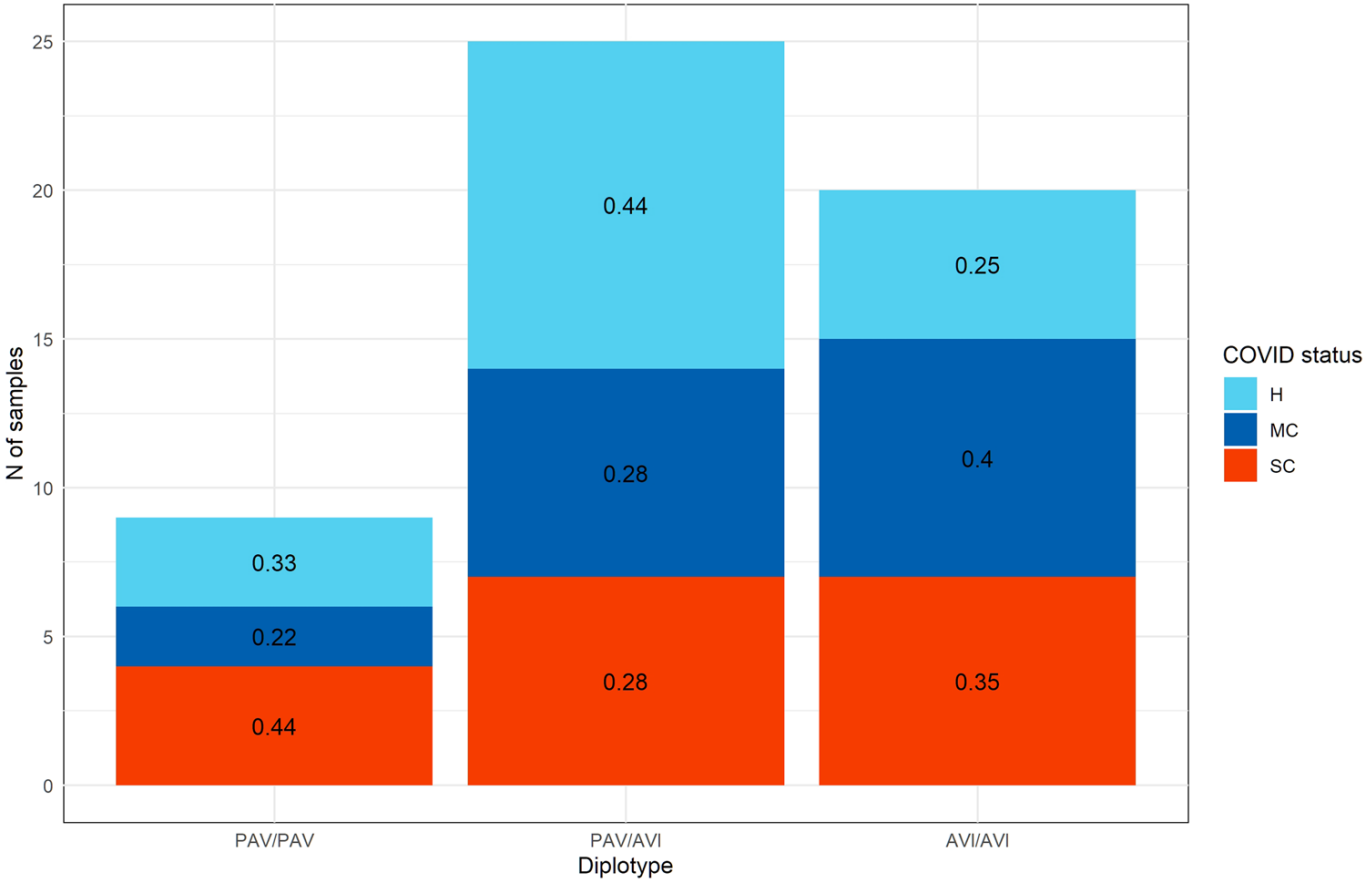
COVID status distribution among TAS2R38 diplotypes.

As any other direct significant interaction between TAS2R38 genotypes and COVID-19-related variables was not detected, the SNPs-related diplotype information was leveraged to find differences among infection status categories. Indeed, when computing COVID-19 frequencies for each diplotype class, it was detected a noticeable variation of the former among PAV/PAV, PAV/AVI and AVI/AVI groups. In particular, the proportion of samples marked as SC is 44% in PAV/PAV, 28% in PAV/AVI and 35% in AVI/AVI. Sharper variations are noticeable in the MC and H groups, respectively with 22%, 28%, 40% and 33%, 44%, and 25% in PAV/PAV, PAV/AVI and AVI/AVI diplotypes (Fig. 3).

It was tested if the number of PAV and AVI haplotypes in each sample differed for the three pairs of haplotypes using ANOVA test, our results, though, were not above the rejection threshold level (p > 0.05). Additionally, it was created a contingency table summarizing the apportionment of samples among TAS2R38 diplotypes and COVID status, to be tested via Chi-squared test. The result of Chi-squared highlighted no differences in the severity of infection for the different diplotypes. Moreover, no significance was found even considering the occurrences of infected and non-infected samples in PAV/PAV, PAV/AVI, and AVI/AVI groups, using Fisher exact test. Altogether, these results highlight how no detectable implication, in our dataset, exists between infection and haplotypes configurations of TAS2R38 SNPs.

Lastly, non-genetic variables were tested to assess whether, if any, differences may exist among PROP perception and COVID-19 infection status and dysgeusia. ANOVA reported non-significant results for both tests, uncoupling PROP-related phenotype to SARS-CoV-2 infection.

## Conclusions

In the present study, for the first time, the frequency of TASR38 PAV and AVI alleles in COVID-19 patients with different severity of the diseases and healthy subjects has been investigated. The 54 individuals included in the study only carried the two most common haplotypes of TAS2R38, confirming that limiting our focus on Caucasian samples helped narrowing down haplotype heterogeneity. Our analysis established, once again, the strong relationship between TAS2R38 diplotypes and PROP perception, highlighting the quality of the sampling procedure and data collection. Nevertheless, our investigation could not point out at a significant relationship between SNPs responsible for PROP bitterness and presence/severity of SARS-CoV2 infection, as previous studies suggested when looking at taster and non-taster phenotypes. Our results uncouple the direct genetic contribution of rs10246939, rs1726866 and rs713598 on COVID-19, calling for caution when proposing a treatment based on TAS2R38 phenotypes. However, these data apply to individuals without systemic diseases and who are not under treatment with pharmacological agents.
